# The emerging role of Nrf2 in dermatotoxicology

**DOI:** 10.1002/emmm.201303797

**Published:** 2014-02-12

**Authors:** Nguan S Tan, Walter Wahli

**Affiliations:** 1 School of Biological Sciences, Nanyang Technological UniversitySingapore City, Singapore; 2 Institute of Molecular and Cell Biology, A*STARSingapore City, Singapore; 3 Lee Kong Chian School of Medicine, Nanyang Technological University, Singapore General HospitalSingapore City, Singapore; 4 Center for Integrative Genomics, University of LausanneLausanne, Switzerland

## Abstract

The nuclear factor erythroid 2-related factor 2 (Nrf2) is best known for its role in resistance to oxidant stress. In this issue of *EMBO Molecular Medicine*, Nrf2-prolonged genetic activation is shown with devastating effects on skin homeostasis. The study provides novel molecular insights into poison-induced chloracne and metabolizing acquired dioxin-induced skin hamartomas or MADISH.

Skin helps insulate the body and protect it from mechanical stress, excessive water loss, and pathogens. Interestingly, many noxious substances entering the body through the skin or even systemically are metabolized at least partly in keratinocytes. Not surprisingly, poisoning is often associated with skin lesions and diverse skin reactions.

In this context, chloracne is one of the most sensitive biological indicators of occupational or environmental exposure to certain highly toxic halogenated aromatic hydrocarbons. Such substances are often trace contaminants produced during the synthesis of industrial chemicals, such as herbicides. Because low concentrations of dioxins are widely distributed throughout the environment and are both persistent and bioaccumulated, millions of people are exposed to dioxin and dioxin-like compounds, primarily through contaminated food. Poison-induced chloracne appears regardless of whether chemical exposure has occurred via skin contact, inhalation, or ingestion. One of the most potent inducers of chloracne is 2,3,7,8-tetrachlorodibenzo-p-dioxin (TCDD), and chloracne is the only skin disorder consistently reported to be associated specifically with TCDD and other herbicides (Ma, 2013). The clinical features of chloracne include multiple closed comedones that evolve into deep cysts. As toxicity increases, the posterior neck, trunk, and extremities may be involved, while palms and soles, which have no sebaceous glands (SGs), are spared. Thus, based on clinical findings alone, it can be difficult for physicians to differentiate chloracne from some forms of *acne vulgaris*. TCDD became notorious as a contaminant of Agent Orange, an herbicide used during the Vietnam War. More recently, much was learned about TCDD lesions from the 2004 assassination attempt of the 3rd president of the Ukraine, Viktor Yushchenko. He was poisoned by exposure to TCDD at a single oral dose that was 5 million-fold greater than the accepted daily exposure limit. Yushchenko's skin lesions included hamartomas with a complete involution of SGs and cysts (Saurat *et al*, 2012). These lesions were called “metabolizing acquired dioxin-induced skin hamartomas” (MADISH). These hamartomas accumulate TCDD and express high levels of the TCDD-metabolizing enzyme cytochrome P450 1A1; thus, they represent a detoxification structure that is thought to reduce the systemic toxicity of TCDD. TCDD affects keratinocyte differentiation *in vitro* (Sutter *et al*, 2011), and TCDD-mediated reactive oxygen species (ROS) production is a critical step in TCDD-accelerated keratinocyte differentiation (Kennedy *et al*, 2013). TCDD also induces changes in the pilosebaceous unit: The epidermis and infundibulum undergo prominent hyperplasia and hyperkeratosis, while SGs and sweat glands become dysfunctional and are replaced by keratinizing cells, resulting in cyst/hamartoma formation (Ju *et al*, 2009; Saurat *et al*, 2012).

This strongly resembles the pathology of patients with chloracne/MADISH at both the histological and molecular levels.

The work by Schäfer *et al* (2014), published in this issue of *EMBO Molecular Medicine*, offers novel insights into the molecular basis of MADISH. It shows that prolonged genetic activation of the nuclear factor erythroid 2-related factor 2 (Nrf2) in keratinocytes has a devastating impact on skin homeostasis and leads to abnormalities in the pilosebaceous unit. This was unexpected because a major function of Nrf2 is its role in resistance to oxidant stress. Nrf2 is a transcription factor that senses radiation-and chemical-induced oxidative and electrophilic stresses; it controls a battery of defensive genes encoding antioxidant proteins and detoxifying enzymes, such as NAD(P)H:quinone oxidoreductase 1 (NQO1) and glutathione-S-transferases. Accordingly, Nrf2-null mice show increased susceptibility to a broad range of chemical toxicities and to disease conditions associated with oxidative pathology, while pharmacological activation of Nrf2 by chemoprotective agents protects against oxidative damage (Ma, 2013). However, Nrf2 may also protect cancer cells from chemotherapeutic drugs and thereby facilitate tumor cell survival (Jaramillo & Zhang, 2013).

In the study by Schäfer *et al* (2014), keratinocyte-specific expression of constitutively active Nrf2 in mice led to SG enlargement and seborrhea with hyperkeratosis and acanthosis of the hair follicle infundibuli and interfollicular epidermis, thus replicating some characteristics of non-inflammatory acne in humans. As these mice aged, they exhibited a MADISH phenotype characterized by the dilatation of infundibuli and developed larger keratinized skin cysts combined with the loss of SGs. This strongly resembles the pathology of patients with chloracne/MADISH at both the histological and molecular levels. This phenotype was initiated by upregulation of epigen (Epgn), a growth factor and EGFR ligand that was identified in the study as an Nrf2 target. Epgn is expressed in undifferentiated sebocytes, and Epgn transgenic mice develop enlarged SGs due to expansion of basal, undifferentiated cells in the gland in response to Epgn overexpression (Dahlhoff *et al*, 2010). SG enlargement was accompanied by increased expression of the nuclear receptor peroxisome proliferator-activated receptor γ (PPARγ) (Schäfer *et al*, 2014), which is important for sebocyte differentiation and stimulation of a subset of lipogenic genes for lipid storage in these cells (Dozsa *et al*, 2013).

The study also identified previously unknown functions of two Nrf2 target genes that encode the secretory leukocyte peptidase inhibitor (*Slpi*) and small proline-rich protein 2 (*Sprr2d*) in the pilosebaceous unit. These two proteins contribute to the ROS-protective and anti-inflammatory activities of Nrf2 because Sprrs are potent scavengers of ROS and Slpi has antimicrobial activities (Schäfer *et al*, 2012). However, prolonged Nrf2-induced overexpression of these two proteins led to infundibulum acanthosis and hyperkeratosis and ultimately to cyst formation. It also caused corneocyte fragility and impaired desquamation and, consequently, to alterations in the epidermal lipid barrier, inflammation, and overexpression of mitogens that induce keratinocyte hyperproliferation (Schäfer *et al*, 2012, 2014). Clinically, these observations suggest a novel function of NRF2 and its targets *SPRR2D* and *SLPI* in MADISH pathogenesis.

Guided by the MADISH-like phenotype observed in their Nrf2 transgenic mice, Schäfer *et al* (2014) treated human keratinocytes, which are the cells in MADISH patients that are responsible for cyst formation, with TCDD. This resulted in upregulation of *NRF2* and its targets *SPRR2D*, *SLPI,* and *EPGN* via activation of the aryl hydrocarbon receptor (AHR, TCDD receptor), which in turn stimulated its target genes *CYP1A1* and *CYP1B1*. Both AHR and NRF2 are required for the TCDD-mediated activation of *SPRR2D*, *SLPI,* and *EPGN,* revealing a novel AHR-NRF2 signaling axis in keratinocytes (Fig 1). Supporting the idea that the Nrf2 transgenic mouse is a clinically relevant model for human MADISH-like skin disease, Schäfer *et al* (2014) also detected strong expression of SPRR2D, SLPI, EPGN, and the classical NRF2 target, NQO1, in the epidermis and cysts epithelium of MADISH patients. Together, these novel data provide insights into the molecular events that occur in keratinocytes after TCDD and unveil a novel TCCD-AHR-NRF2-SPRR2/SLPI/EPGN axis in the pathogenesis of MADISH.

**Figure 1:**
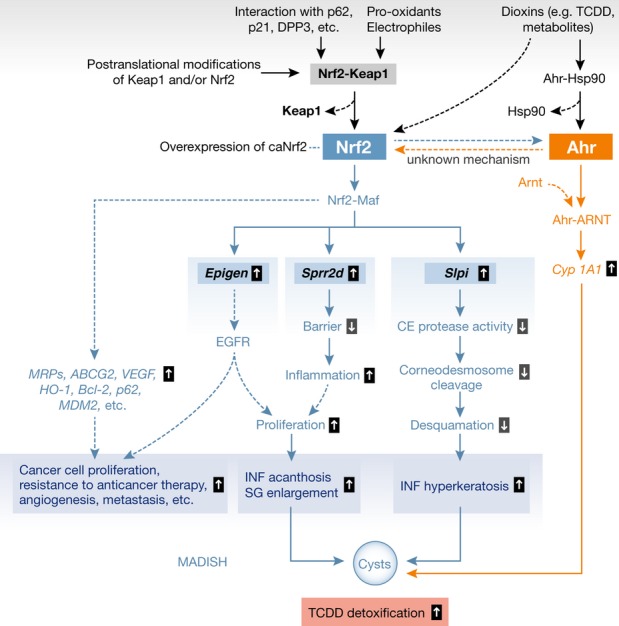
In the absence of stress, Nrf2 forms an inactive complex with Keap1, which is sequestered in the cytoplasm. Oxidative or electrophilic stress causes this complex to interact with regulatory proteins, while post-translational modifications can result in dissociation of Nrf2 from Keap1. Free Nrf2 migrates to the nucleus where it interacts with a small Maf protein to form a complex that transactivates target genes that encode antioxidant and cytoprotective proteins. In hair follicle infundibuli, Nrf2 activation stimulates *Epigen, Sprr2d,* and *Slpi*. Epgn stimulates proliferation of infundibular keratinocytes via EGFR signaling, while Sprr2d upregulation weakens the epidermal barrier. The latter leads to inflammation and enhanced keratinocyte proliferation that results in SG enlargement and acanthosis. Slpi upregulation leads to decreased desquamation, which contributes to hyperkeratosis. Nrf2 signaling is also frequently increased in cancer following events such as mutation of Nrf2 and Keap1, protein–protein interactions with Keap1, and low Keap1 expression. This increases nuclear Nrf2 levels and thus its transcriptional activity. The involvement of Epgn in cancer is not well understood. When the dioxin receptor Ahr is activated, it stimulates the expression of detoxifying genes such as *Cyp1A1* and functionally interacts with Nrf2 to enhance its signaling. In MADISH, it is thought that Nrf2 and Ahr cooperate to ameliorate dioxin detoxification. AHR activates Nrf2 transcription and vice versa in other cell types.

The early molecular events that initiate the MADISH pathogenesis are not well understood, which would require analysis of the epidermis from patients exposed to chloracnegens at an early stage. Such biological samples are not readily available, since patients only present for treatment when their symptoms appear. At this stage, the observable alterations are secondary to the initial changes. Further, the early events that occur in human skin cannot be studied in TCDD-treated mice because they do not develop a skin hamartoma phenotype, possibly because TCDD is metabolized differently in rodents than in humans or because the biological response to TCDD differs in the two.

Clinically, these observations suggest a novel function of NRF2 and its targets *SPRR2D* and *SLPI* in MADISH pathogenesis.

Nrf2 signaling is complex (Fig 1). Under basal conditions, Nrf2 is associated with Keap1, which brings Nrf2 into the Keap1-Cul3-E3 ubiquitin ligase and results in Nrf2 degradation. Oxidative stress and electrophiles weaken the Nrf2-Keap1 complex, and Nrf2 accumulates in the nucleus, where it dimerizes with members of the small Maf family transcription factors and binds to antioxidant response elements (AREs) within the regulatory regions of a wide variety of target genes (Jaramillo & Zhang, 2013). Furthermore, the Nrf2-Keap1 complex is controlled by its crosstalk with other proteins, such as the substrate adaptor sequestosome 1 protein, cyclin-dependent kinase inhibitor 1, and dipeptidyl peptidase 3. It is unknown whether these interactions, which occur in cancer cells, also control Nrf2 activity in keratinocytes during MADISH pathogenesis and whether they are controlled by TCDD-AHR signaling.

While it is tempting to propose that Nrf2 and/or the Nrf2-Keap1 complex are potential targets for therapeutics that reduce MADISH progression, such Nrf2-based drugs will likely impede TCDD detoxification in situations in which it would be preferable to activate Nrf2 to increase detoxification. These questions require further investigation, precluding a simple conclusion about the role of Nrf2 in the etiology of chloracne.
